# Model-based prediction of nanoparticle and dissolved form ratios using total concentration data: a case study of SNB-101

**DOI:** 10.3389/fphar.2025.1556618

**Published:** 2025-08-21

**Authors:** Jinha Park, Soo Hyeon Bae, Sangil Jeon, Young Hwan Park, Dong Cheol Lee, Seunghoon Han

**Affiliations:** ^1^ AIMS BioScience, Co., Ltd., Seoul, Republic of Korea; ^2^ Department of Pharmacology, College of Medicine, The Catholic University of Korea, Seoul, Republic of Korea; ^3^ SN BioScience, Inc., Seongnam-si, Gyeonggi-do, Republic of Korea

**Keywords:** SN-38, nanoparticle drug delivery, pharmacokinetic modeling, tumor targeting, enhanced permeability and retention effect

## Abstract

**Introduction:**

Irinotecan (CPT-11), a topoisomerase I inhibitor, serves as a prodrug for SN-38, its active metabolite with significantly higher cytotoxic potency. Despite its clinical efficacy, irinotecan’s therapeutic potential is limited by low fraction of conversion to SN-38, inefficient tumor targeting, and dose-limiting toxicities such as diarrhea and neutropenia. Nanoparticle-based formulations, such as SNB-101, offer a promising solution by encapsulating irinotecan and SN-38, enhancing solubility, improving drug delivery efficiency, and reducing systemic toxicity through tumor-specific accumulation via the enhanced permeability and retention (EPR) effect.

**Methods:**

This study aimed to develop a pharmacokinetic (PK) model to differentiate between nanoparticle (NP) and dissolved (S) forms of irinotecan and SN-38 using total plasma concentration data from a Phase I clinical trial of SNB-101 (NCT04640480). The 11-compartment model incorporated prior knowledge of dissolved irinotecan PK and newly observed clinical data to characterize NP-to-S transitions and their respective contributions to total drug exposure.

**Results:**

Results revealed that SNB-101 is predominantly predicted to deliver SN-38 in its nanoparticle form, with NP-SN-38 contributing over 80% of total SN-38 exposure. The high exposure to NP-SN-38 correlated with reduced systemic toxicity compared to conventional irinotecan formulations, despite significantly increased total SN-38 levels.

**Discussion:**

This reduced exposure to dissolved SN-38 and irinotecan likely underpins the favorable safety profile observed in dose-escalation studies. This model-based approach underscores the utility of nanoparticle formulations in improving drug delivery and highlights the importance of distinguishing between NP and S forms for accurate efficacy and toxicity predictions. The framework may provide a useful tool for optimizing dose selection and accelerating the clinical development of nanoparticle-based therapeutics.

## 1 Introduction

Irinotecan (CPT-11), a topoisomerase I inhibitor, has been widely used as a first-line treatment for metastatic colorectal cancer since its approval in 1996 (Kciuk et al., 2020). The therapeutic effect of irinotecan is manifested when it is converted into an active metabolite, SN-38 (7-ethyl-10-hydroxycamptothecin), which has 100 to 1,000 times more potent cytotoxic activity (Kciuk et al., 2020; Takeba et al., 2007; Lee et al., 2019; Kawato et al., 1991). However, SN-38 cannot be administered directly because it has very low solubility in water, which severely limits the bioavailability of the drug (Paoluzzi et al., 2004; Haaz et al., 1997). Therefore, irinotecan acts as a prodrug that delivers SN-38 in a more soluble and clinically viable form. The clinical utility of irinotecan is well known for improving survival rates in patients with advanced colorectal cancer, and its importance in oncology has been emphasized as it has been included in various chemotherapy regimens.

However, the clinical application of irinotecan is limited by systemic toxicity and the inefficiency of delivering SN-38 to tumors. Only a small fraction (approximately 2%–8%) of irinotecan is converted to SN-38, which is further metabolized to the inactive conjugate SN-38 glucuronide (SN-38G) through glucuronidation by UGT1A1 and UGT1A9 (Rothenberg et al., 1993; Meyer-Losic et al., 2008; Mathijssen et al., 2001). On the other hand, a significant portion of irinotecan is metabolized into less active metabolites, for example, 7-ethyl-10-[4-N-(5-aminopentanoic acid)-1-piperidinyl] carbonyloxycamptothecin (APC) and 7-ethyl-10-[4-amino-1-piperidino] carbonyloxycamptothecin (NPC) (Santos et al., 2000). These metabolic pathways limit the effective utilization of SN-38 for therapeutic action and contribute to the clinical ineffectiveness of irinotecan. In addition, the low targeting ability of irinotecan causes serious side effects, including dose-limiting toxicities such as severe diarrhea and neutropenia.

Nanoparticle-based drug delivery systems have emerged as a promising solution to overcome these limitations. Nanoparticles overcome the low water solubility and low bioavailability by directly encapsulating SN-38, allowing irinotecan to be administered directly without the need for a prodrug. Nanoparticles also take advantage of enhanced permeability and retention (EPR) effects to preferentially accumulate in tumor tissue due to the abnormal vasculature and compromised lymphatic drainage of the tumor microenvironment (Wu, 2021). This targeted delivery increases the concentration of SN-38 in the tumor, thereby enhancing its efficacy while minimizing systemic exposure and reducing toxicity. The EPR effect also suggests that the nanoparticle (NP) form of the active pharmaceutical ingredient (API) may be more directly related to efficacy because it accumulates preferentially in tumors. In contrast, the dissolved (S) form may be more associated with systemic toxicity, as off-target effects such as neutropenia and severe diarrhea are often associated with non-specific systemic exposure.

This distinction is critical to optimizing treatment outcomes, but early drug development typically relies on total concentration data combining NP and S forms due to practical considerations. Although validated analytical methods, such as UPLC-MS/MS combined with solid-phase extraction, are available to differentiate NP and S forms, implementing these advanced methods requires significant investment in resources, time, and cost (Desai, 2012). Specifically, nanoparticles can rapidly interact with plasma proteins to form protein corona, which can alter their physicochemical properties. In addition, the inherent variability in the size, shape, and surface properties of nanoparticles requires a highly customized analytical approach. In addition, nanoparticles can dissolve or aggregate during sample preparation, distorting the original NP-to-S ratio. These challenges collectively increase the complexity, duration, and cost associated with establishing reliable analytical methods capable of differentiating NP and S forms. Consequently, early-phase drug development, particularly in biotechnology companies where cost efficiency and rapid progression are crucial, frequently utilizes simpler and more cost-effective methods based on measuring total drug concentrations. This practical limitation underscores the need for alternative strategies to estimate NP-to-S ratios from readily accessible total concentration data.

To address these issues, integrating existing PK knowledge of S form provides a model-based framework for estimating the NP-to-S ratio using total concentration data. By leveraging the known PK properties of the S form, total concentration data can be deconvoluted to predict the individual contributions of NP and S forms. This approach allows for more accurate characterization of the efficacy and toxicity profiles of nanoparticle formulations, even in the absence of direct NP/S-specific concentration measurements. This study proposes a model-based method to predict the ratio of NP to S in SNB-101 (SN BioScience, Inc. Seongnam-si, Gyeonggi-do, Republic of Korea), a polymeric nanoparticle formulation with a mean particle size of approximately 110 nm. SNB-101 consists of irinotecan and SN-38 encapsulated in biocompatible block co-polymers at a 1:1 molar ratio. Unlike conventional irinotecan formulations, SNB-101 enables direct, high-dose SN-38 administration (5–50 mg/m^2^). Due to the highly non-polar nature of SN-38, a more polar component was essential to ensure nanoparticle stability. Irinotecan was identified as optimal for this purpose due to its polarity and *in vivo* conversion to SN-38. Formulation optimization studies determined that a minimum of 50% irinotecan was necessary to achieve adequate stability, resulting in the selected composition. This framework aims to improve the accuracy of efficacy and toxicity prediction to support better decision-making in phase II trials and accelerate the development of nanoparticle-based therapeutics.

## 2 Materials and methods

### 2.1 Clinical trial as the source of observed dataset

This analysis used PK data derived from a completed Phase I clinical trial (NCT04640480) evaluating SNB-101. The dataset included total plasma concentration measurements of irinotecan, SN-38, and SN-38 glucuronide (SN-38G) obtained following intravenous administration of SNB-101. Subjects were assigned to seven dosing cohorts, with SN-38 doses ranging from 5 mg/m^2^ to 50 mg/m^2^. The distribution of subjects across cohorts for Cycle 1 is summarized in Table 1.

**TABLE 1:** Cohort information.

Cohort	*N*	Irinotecan dose (mg/m^2^)	SN-38 dose (mg/m^2^)	Sex (M/F)	Age (yr)	Weight (kg)
1	1	8	5	1/0	60	70
2	1	16	10	1/0	62	72
3	1	32	20	1/0	58	68
4	5	48	30	3/2	65.0 ± 1.4	75.0 ± 1.4
5	7	64	40	4/3	63.0 ± 2.0	73.0 ± 2.0
6	3	72	45	2/1	59.0 ± 0.8	70.0 ± 0.8
7	3	80	50	2/1	62.0 ± 0.8	73.0 ± 0.8

The trial enrolled adult patients with advanced solid tumors. Eligible participants had histologically or cytologically confirmed malignancies that were refractory to standard therapies or for which no standard treatment existed. Key inclusion criteria included an Eastern Cooperative Oncology Group (ECOG) performance status of 0 or 1, adequate bone marrow, liver, and renal function, and a life expectancy of at least 3 months. Exclusion criteria encompassed active infections, significant cardiovascular disease, and prior treatment with topoisomerase I inhibitors.

**FIGURE 1:**
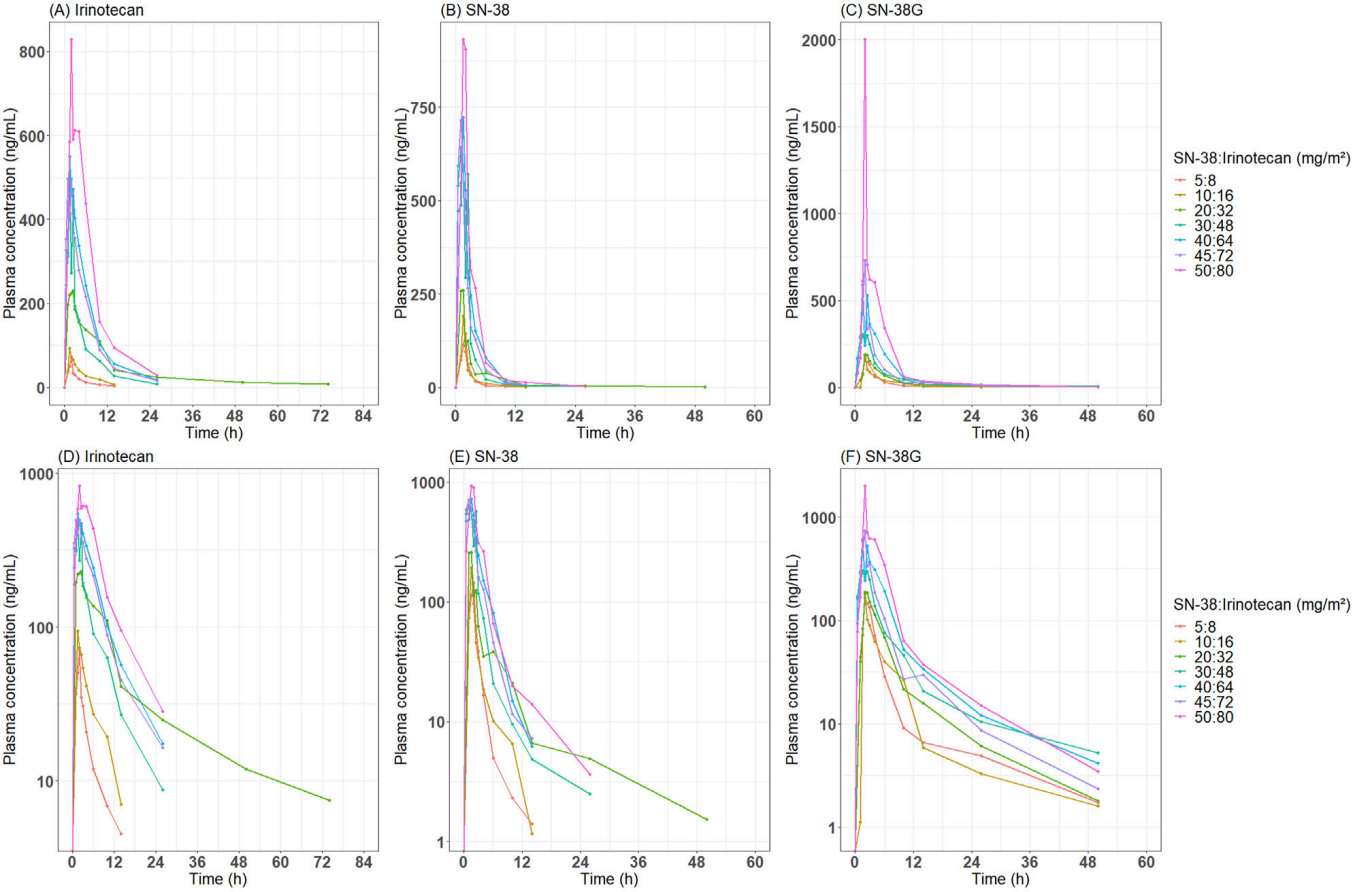
Observed time-concentration profile of irinotecan, SN-38 and SN-38G after administration of SNB-101. Median concentration-time profiles by dose level are presented for **(A)** irinotecan, **(B)** SN-38, and **(C)** SN-38 glucuronide on an arithmetic scale, and **(D)** irinotecan, **(E)** SN-38, and **(F)** SN-38 glucuronide on a semi-logarithmic scale.

Blood samples were collected during Cycle 1 at pre-dose (0 h), and at 0.5, 1, 2, 4, 6, 8, 10, 12, 24, 36, 48, 72, 96, 120, 144, and 169.5 h post-dose. These extensive time points provided a comprehensive characterization of the PK for both irinotecan and its metabolites during the first cycle of administration. As is common in early-phase trials for nanoparticle formulations, the measured total concentrations represented the combined contributions of NP and S forms. This limitation necessitated the development of a model-based framework to estimate the NP-to-S ratios within the total concentration data. The overall time–concentration profile of irinotecan, SN-38, and SN-38G is presented in Figure 1.

### 2.2 Model development

The PK model was developed to characterize the time–concentration profiles of irinotecan, SN-38, and SN-38G and to estimate their contributions to the overall plasma concentration in both forms. The model was built by integrating the formulation-specific characteristics of SNB-101, prior PK knowledge from published studies (for S form irinotecan and SN-38) (Ahn et al., 2010; Minichmayr et al., 2021), and observed clinical data.

Based on existing development data for SNB-101, it is known that the ratio of the presence of irinotecan and SN-38 in the NP form and S form in long-term stored drug products is 0.15:0.85 and 0.98:0.02, respectively, and these proportions were incorporated into the dosing information in the model. Since SNB-101 was administered intravenously, the four conceptual analytes (NP-irinotecan, S-irinotecan, NP-SN-38, and S-SN-38) were each modeled using their own separate central compartments. (e.g., NP-irinotecan is administered to the NP-irinotecan central compartment). Considering that SNB-101 contains irinotecan and SN-38 co-encapsulated within the same nanoparticle, it was assumed that NP-irinotecan and NP-SN-38 exhibit similar pharmacokinetic behavior. Therefore, their disposition characteristics (e.g., number of compartments, intercompartmental clearance) were modeled using shared parameter values.

The structural model was based on the compartmental model for S-irinotecan (administered as irinotecan) that was previously established (Minichmayr et al., 2021). This model fixed the structure of S-irinotecan (a three-compartment model with first-order elimination) and parameter values to those in the literature. Total plasma irinotecan concentration was set as the sum of NP-irinotecan and S-irinotecan concentrations. Notably, the PK profile of irinotecan following SNB-101 administration was assumed to be driven primarily by NP-irinotecan, in contrast to conventional formulations. The release of irinotecan from the nanoparticles was reflected in the model by drug movement from the central compartment of NP-irinotecan to the central compartment of S-irinotecan, which was the only elimination route for NP-irinotecan. A multi-compartmental structure was tested for NP-irinotecan during model development to best capture its disposition characteristics.

The total plasma concentration of SN-38 was assumed to be the sum of NP-SN-38 and S-SN-38. The proportion of SN-38 derived from irinotecan metabolism was fixed at 0.03 (3%), and S-irinotecan was set to be converted to S-SN-38 within the central compartment of each analyte. Any changes in the total SN-38 concentration that could not be explained by irinotecan metabolism were attributed to the administration of NP-SN-38 administration. The only elimination pathway for NP-SN-38 was assumed to be its release into S-SN-38. Furthermore, the elimination of SN-38 was assumed to occur exclusively through its metabolism to SN-38G within the central compartment of S-SN-38. A multiple compartmental model was evaluated for both NP-SN-38 and S-SN-38. Since SN-38G exists only in dissolved form, its observed concentrations could be directly incorporated into the model. For SN-38G, single-compartment or two-compartment models were evaluated during model development.

All PK processes were assumed to follow first-order kinetics, and between-subject variability (BSV) was estimated (log-normal distribution) for as many variables as possible. A separate proportional error model was introduced for each analyte. The model was selected based on the visual evaluation using goodness-of-fit (GoF) plots and the change in objective function value (OFV) to ensure statistical rigor. The nested models were compared using the likelihood ratio test, and at least 3.84 (p < 0.05, 1 degree of freedom) or greater was considered statistically significant. This criterion was used to justify the inclusion of additional variables. Forward selection and backward elimination were applied during the covariate analysis process to ensure that only statistically significant and clinically meaningful covariates were retained. OFV was used as the primary metric for model improvement, but model robustness and convergence were equally emphasized. Models with high ETA shrinkage, large condition numbers, or poor convergence diagnostics were modified or excluded regardless of OFV improvement.

The nonlinear mixed-effects modeling and simulation of SNB-101 were performed using NONMEM version 7.5 (Icon Development Solutions, Ellicott City, MD, USA).

### 2.3 Final model evaluation and simulation

The goal of model development in this study was to predict the PK properties of the NP form and S form by separating them from the total concentration using existing information. Therefore, we determined that bootstrap, which is a commonly used evaluation method, is not an appropriate evaluation method because it does not evaluate whether the parameter estimate is robust despite changes in the population composition. Instead, the validity of the model was evaluated by comparing the total concentration change by time period as the sum of the predicted NP and S forms through a single-dose-based visual predictive check. To this end, 1,000 simulated datasets were generated for each dose, and the median and 90% prediction interval were obtained for each sampling timepoint. In addition, the concentration changes of NP form and S form of each analyte over time were predicted for each subject enrolled. The simulation was performed to see the typical values for the maximum exposure (maximum plasma concentration, *C*_max_) and total exposure over the dosing interval (area under the time-concentration curve until 336 h post-dose, *AUC*_0-336_) including extended dose levels (up to 100 mg/m^2^ as SN-38).

## 3 Results

### 3.1 Final PK model and evaluation outcomes

**FIGURE 2:**
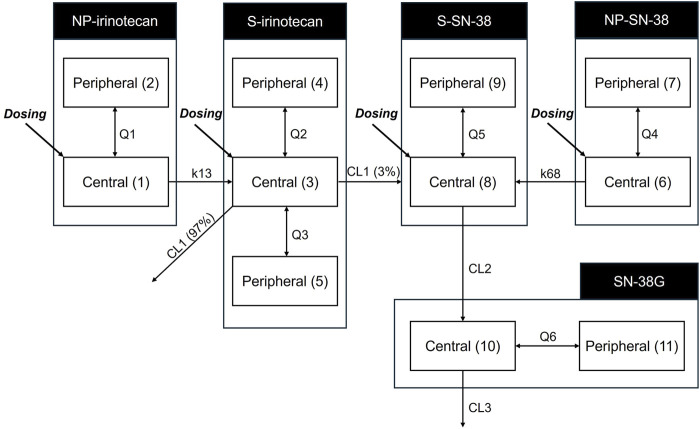
Pharmacokinetic model structure for irinotecan, SN-38 and SN-38G considering both nanoparticle form and dissolved form. The model differentiates between nanoparticulate (NP) and dissolved (S) forms of irinotecan and SN-38. The numbers in brackets indicate the number of compartments. CL1, clearance of dissolved irinotecan (L/h); CL2, clearance of dissolved SN-38 (L/h); CL3, clearance of SN-38 glucuronide (L/h); K13, rate constant of nanoparticulated irinotecan to dissolved irinotecan (/h); K68, rate constant of nanoparticulated SN-38 to dissolved SN-38 (/h); NP, nanoparticulated form; Q1, intercompartmental clearance of nanoparticulated irinotecan (L/h); Q2, intercompartmental clearance of dissolved irinotecan (L/h); Q3, intercompartmental clearance of dissolved irinotecan (L/h); Q4, intercompartmental clearance of nanoparticulated SN-38 (L/h); Q5, intercompartmental clearance of dissolved SN-38 (L/h); Q6, intercompartmental clearance of SN-38 glucuronide (L/h); S, dissolved form; SN-38G, SN-38 glucuronide.

An 11-compartment PK model was developed to distinguish between the NP and S forms of SNB-101 analytes including irinotecan and SN-38 (Figure 2). Because of the presence of all observations for irinotecan, SN-38, and SN-38G, albeit in total concentration, a two-compartment model could be successfully established for NP-irinotecan, NP-SN-38, S-SN-38, and SN-38G, while retaining the previously known three-compartment model for S-irinotecan. All doses were well fit without including PK parameter-specific fixed effects for each dose level, indicating dose proportionality. Despite the small number of subjects and data, we were able to obtain relatively reliable population PK parameters and estimate BSVs for some parameters.

The central volume of distribution (V1 = V6) of the NP-form was 54.4 L, which was intermediate between that of S-irinotecan (V3: 68.6 L) and S-SN-38 (V8: 40.3 L). The rate constants for the conversion from NP to S form (k13: 64.4 h^−1^ for irinotecan and k68: 0.712 h^−1^ for SN-38) suggested a much faster release of irinotecan from the nanoparticles (see Table 2 for final parameter estimates). The peripheral compartment volume of S-SN-38 (V9) was estimated to be 491 L, which is indicative of the lipophilic nature of the SN-38. The elimination half-life of S-SN-38 to SN-38G was calculated as 2.2 h which is smaller than previously reported value. A relatively large BSV was observed in the PK parameter of SN-38G, indicating that there may be large interindividual differences in the metabolism of SN-38 to SN-38G and its elimination. The covariate analysis did not identify any statistically significant influencing factors.

**TABLE 2:** Parameter fixed values or estimates in the final model.

Parameter	Description	Estimate	BSV
CL1	Clearance of S-Irinotecan (L/h)	29.7	34.7%
V1 (=V6)	Central volume of distribution of NP-Irinotecan (NP-SN-38) (L)	54.4	40.9%
V2 (=V7)	Peripheral volume of distribution of NP-Irinotecan (NP-SN-38) (L)	287	94.5%
Q1 (=Q4)	Intercompartmental clearance of NP-Irinotecan (NP-SN-38) (L/h)	6.45	
k13	Rate constant of NP-Irinotecan to S-Irinotecan (h^-1^)	64.4	
V3	Central volume of distribution of S-Irinotecan (L)	68.6 FIX	15.4% FIX
V4	Rapid peripheral volume of distribution of S-Irinotecan (L)	67.2 FIX	16.2% FIX
V5	Slow peripheral volume of distribution of S-Irinotecan (L)	127 FIX	29.1% FIX
Q2	Intercompartmental clearance between comp.3 and comp.4 (L/h)	114 FIX	86.1% FIX
Q3	Intercompartmental clearance between comp.3 and comp.5 (L/h)	9.89 FIX	49.5% FIX
k68	Rate constant of NP-SN-38 to S-SN-38 (h^-1^)	0.712	39.6%
V8	Central volume of distribution of S-SN-38 (L)	40.3	
V9 (=V11)	Peripheral volume of distribution of S-SN-38 (SN-38G) (L)	491	
Q5	Intercompartmental clearance of S-SN-38 (L/h)	121	
CL2	Clearance of S-SN-38 to SN-38G (L/h)	167	44.9%
CL3	Clearance of SN-38G (L/h)	16.9	115.9%
Q6	Intercompartmental clearance of SN-38G (L/h)	11.3	
V10	Central volume of distribution of SN-38G (L)	7.84	122.1%
PROP1	Proportional error of Irinotecan	0.21	
PROP2	Proportional error of SN-38	0.416	
PROP3	Proportional error of SN-38G	0.308	

**FIGURE 3:**
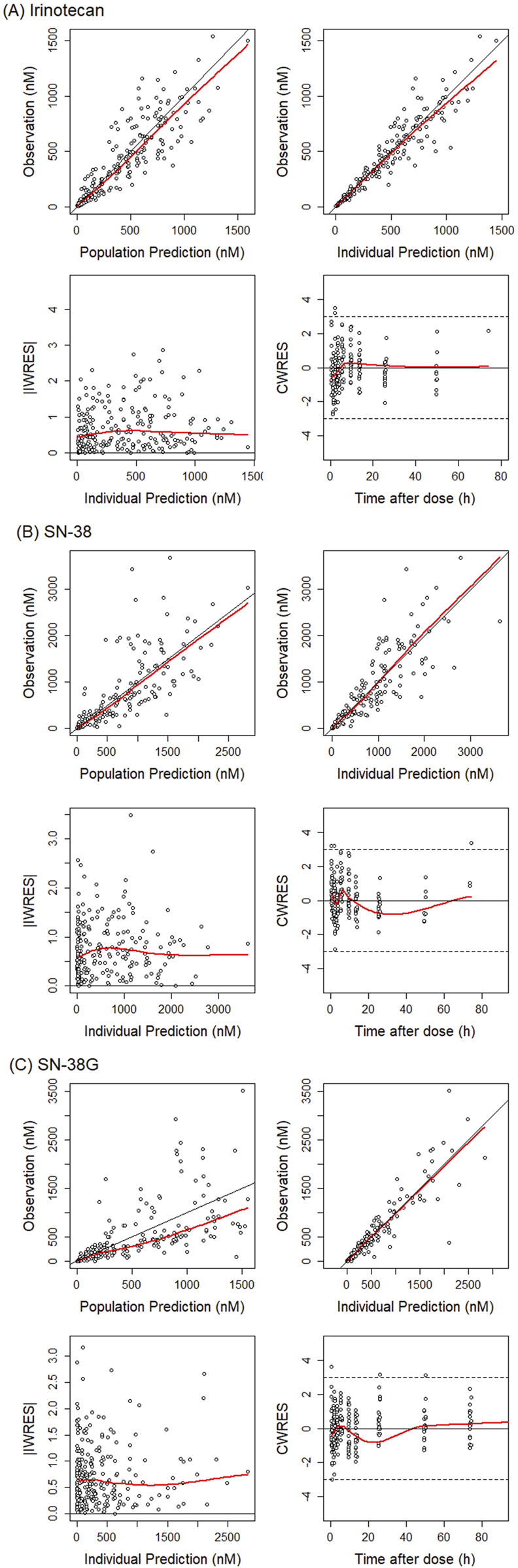
Goodness-of-fit plot for total irinotecan, SN-38 and SN-38G. Basic goodness-of-fit plot are presented for **(A)** irinotecan, **(B)** SN-38, and **(C)** SN-38 glucuronide. The red lines indicate the regression lines. The black lines (y = x or y = 0) are included for reference. The dots represent the observations. iWRES, individual weighted residuals; CWRES, conditional weighted residuals.

**FIGURE 4:**
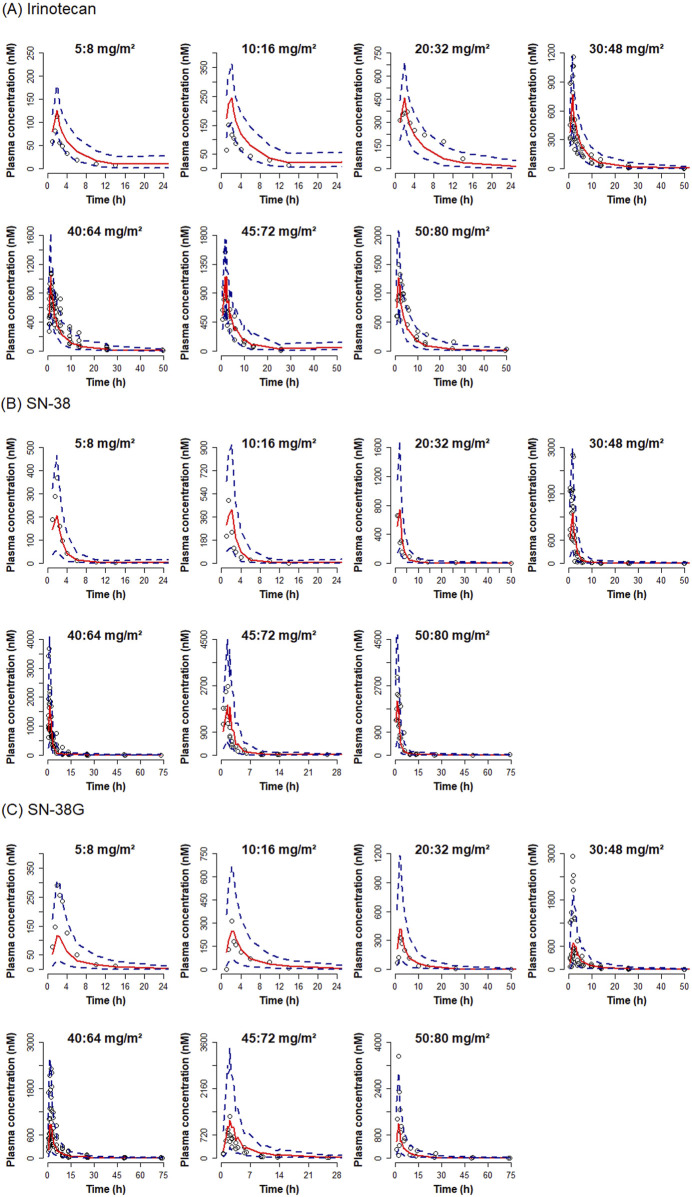
Visual predictive check plot for total irinotecan, SN-38 and SN-38G by dose. Visual predictive check plots are presented for **(A)** irinotecan, **(B)** SN-38, and **(C)** SN-38 glucuronide. Red solid lines represent the medians of simulated concentrations. Blue dashed lines indicate 5% and 95% prediction intervals. Observed concentrations are shown as dots.

**FIGURE 5:**
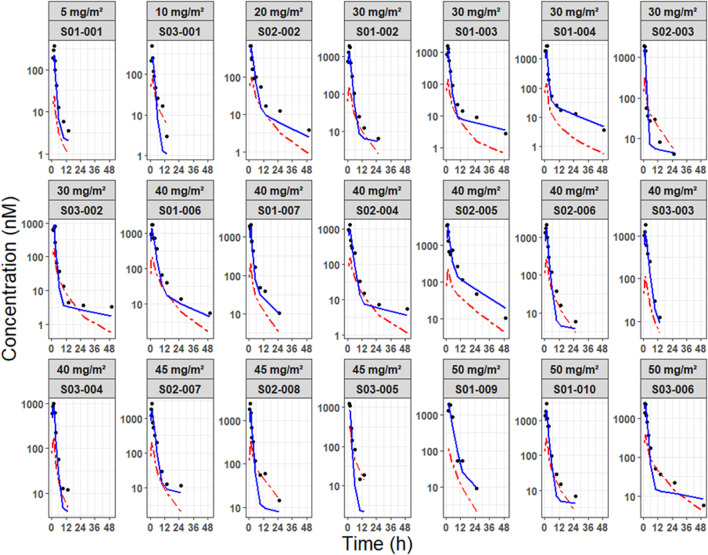
Individual prediction plots of NP-SN-38 and S-SN-38. Predicted concentration-time profiles of SN-38 are differentiated by nanoparticulate form (blue solid line) and dissolved form (red dashed line), with observed concentrations represented as dots. Profiles are stratified by dose level and individual subject.

The GoF plot (Figure 3) confirmed that the observed concentrations for all analytes were evenly distributed around the line of equality. Furthermore, the distribution of residuals showed no systematic bias with respect to the predicted values and time. The overall trend of the predicted intervals from the VPC results was in good agreement with the observed values for each dose and per analyte, indicating good predictive performance of the model at the total concentration level for both NP and S forms combined (Figure 4). Based on these results, predictions could be made about how the NP and S forms of SN-38 (the active substance) would change over time in each individual, with the NP-form expected to predominate throughout the dosing interval (Figure 5).

### 3.2 Simulation findings

The predicted analyte-specific and form-specific *C*_max_ and *AUC*_0-336_ values for each dose are presented in Table 3. The PK of all analytes following SNB-101 administration was linear and dose-proportional, which tended to be consistent with the simulation results. The *C*_max_s were 0.0456, 10.5, 16.5, and 2.35 ng/mL per mg/m^2^ dose for NP-irinotecan, S-irinotecan, NP-SN-38, and S-SN-38, respectively. The *AUC*_0-336_ values were 0.0688, 53.9, 40.5, and 9.87 ng·h/mL per mg/m^2^ dose in the same order. Based on these results, the ratio of *C*_max_ values of NP form to S form was expected to be 0.434% for irinotecan and 70.2% for SN-38, and the proportion of total exposure (*AUC*_0-336_) attributable to the NP form was expected to be approximately 0.127% and 80.4% for irinotecan and SN-38, respectively.

**TABLE 3:** Expected single-dose exposure to nanoparticle or dissolved form irinotecan and SN-38.

Dose of SN-38: irinotecan (mg/m^2^)	NP-irinotecan		S-irinotecan		NP-SN-38		S-SN-38	
	*C*_max_ (ng/mL)	*AUC*_0-336_ (ng·h/mL)	*C*_max_ (ng/mL)	*AUC*_0-336_ (ng·h/mL)	*C*_max_ (ng/mL)	*AUC*_0-336_ (ng·h/mL)	*C*_max_ (ng/mL)	*AUC*_0-336_ (ng·h/mL)
10:16	0.73	1.10	168.72	861.98	164.95	404.73	23.53	98.68
20:32	1.46	2.19	337.45	1723.97	329.91	809.46	47.06	197.37
30:48	2.19	3.29	506.17	2,585.95	494.86	1,214.20	70.59	296.05
40:64	2.92	4.38	674.88	3,447.92	659.82	1,618.94	94.12	394.73
50:80	3.65	5.48	843.62	4,309.93	824.79	2023.68	117.65	493.42
60:96	4.38	6.58	1,012.35	5,171.90	989.75	2,428.45	141.19	592.10
70:112	5.11	7.67	1,181.09	6,033.90	1,154.68	2,833.14	164.71	690.78
80:128	5.84	8.77	1,349.82	6,895.87	1,319.64	3,237.85	188.24	789.46
90:144	6.56	9.86	1,518.49	7,757.87	1,484.61	3,642.60	211.77	888.15
100:160	7.29	10.96	1,687.23	8,619.83	1,649.53	4,047.32	235.31	986.83

## 4 Discussion

The present study successfully developed a PK model to characterize the behavior of NP and S forms of irinotecan and SN-38 following administration of SNB-101. The model employed previously established PK parameters from the literature for S-irinotecan, enabling prediction of the expected plasma concentrations of S-irinotecan. Consequently, discrepancies between the predicted concentrations of S-irinotecan and the measured total irinotecan levels were attributed to nanoparticle irinotecan (NP-irinotecan). This approach enabled the estimation of the conversion parameters governing NP-irinotecan release into S-irinotecan. Furthermore, because S-SN-38 is generated through the metabolism of S-irinotecan, and in minimal and delayed amounts, and the administered SNB-101 contained negligible amounts of S-SN-38, the initially high total SN-38 concentrations were predominantly attributed to NP-SN-38. Furthermore, the formation of SN-38G is exclusively derived from S-SN-38, thereby enabling the model to estimate the clearance of S-SN-38 directly based on observed SN-38G concentrations. The markedly elevated SN-38G concentrations observed in this study, in comparison to conventional irinotecan formulations, provide substantial evidence for a considerable release of S-SN-38 from NP-SN-38. This modeling approach effectively differentiates the contributions of NP-irinotecan, S-irinotecan, NP-SN-38, and S-SN-38, providing a more profound understanding of how SNB-101 may enhance drug delivery efficiency and potentially reduce systemic toxicity compared to conventional irinotecan.

A key finding of this study was the significant increase in total SN-38 exposure following SNB-101 administration compared to the existing irinotecan formulation. According to the Camptosar® label (USFDA, 2014), the typical *C*_max_ of SN-38, even at high doses (340 mg/m^2^), is approximately 56.0 ng/mL, but in this Phase 1 study, total SN-38 concentrations already exceeded this value at the lowest SNB-101 dose level (>100 ng/mL, Figure 1B). Despite this significant increase in SN-38 exposure, dose escalation to 50 mg/m^2^ of SN-38 (80 mg/m^2^ of irinotecan) was successfully completed without any noticeable safety issues (unpublished in-house data). As SN-38 is well recognized as a major contributor to both efficacy and toxicity in irinotecan therapy (Ramesh et al., 2010; Yu et al., 2023), achieving such high concentrations without significant toxicity strongly supports the model’s prediction that most SN-38 is present in NP form following SNB-101 administration. If SN-38 levels were predominantly in S form, as with conventional irinotecan, severe toxicity would have been unavoidable. Accordingly, the model predicts that at the 50:80 mg/m^2^ SNB-101 dose level, a 493.42 ng·h/mL of *AUC*_0-336_, which is comparable to the *AUC*_0-24_ of 474 ng·h/mL reported for S-SN-38 after high-dose (340 mg/m^2^) irinotecan administration. These results indicate that direct delivery of SN-38 via SNB-101 still results in lower exposure to the dissolved form than when high-dose irinotecan is administered. This reduced exposure to S-SN-38 may explain the favorable safety profile observed at dose escalation and further validates the model’s prediction that NP-SN-38 is the predominant form following SNB-101 administration.

This study also provides further insight into the toxicity mechanisms associated with irinotecan formulations. Following SNB-101 administration, irinotecan is rapidly released, and exposure to S-irinotecan was expected to be similar to that observed with conventional irinotecan. Indeed, conventional irinotecan administered at 125 mg/m^2^ resulted in *AUC*_0-24_ of approximately 10,200 ng·h/mL, whereas the model-predicted *AUC*_0-336_ (mostly *AUC*_0-24_, Figure 1A) for dissolved irinotecan following SNB-101 administration at 50:80 mg/m^2^ (SN-38:irinotecan) was 4,309 ng·h/mL. The ratio of these *AUC*s (42.3%) closely matches the dose ratio (40%), and this dose-proportional trend was maintained through simulations scaled up to 100:160 mg/m^2^, supporting the accuracy of the model predictions. Despite similar levels of exposure to S-irinotecan, a favorable toxicity profile was observed in this dose escalation study compared to administration of the original irinotecan formulation, a finding that supports previous studies suggesting that the dose-limiting toxicity of irinotecan products, including severe diarrhea, is due to other factors such as intestinal metabolism rather than plasma concentration (Kehrer et al., 2001; Gupta et al., 1994).

In nanoparticle drug development, especially during early phases, a model-based approach offers notable advantages. Due to practical limitations in resources and analytical methods, directly distinguishing between NP and S forms can be challenging. In this context, a modeling strategy can provide indirect verification that a nanoparticle formulation is achieving its intended PK goals. Although the NP and S form concentrations presented in this study were not directly observed or clinically confirmed, established PK parameters for dissolved forms were leveraged to indirectly estimate the contribution of NP forms. Such an approach is particularly useful when total drug concentration alone is insufficient to fully describe the true dose-exposure relationship of the API. Consequently, this strategy can support informed dose selection, enhanced safety evaluation, and optimization of therapeutic outcomes. The SNB-101 example presented herein clearly demonstrates how this modeling method successfully predicts the substantial contribution of NP-SN-38 relative to S-SN-38, thus explaining the observed favorable toxicity profile. Additionally, the PK of S-irinotecan were well captured by previously reported PK parameters. Overall, these findings emphasize the value of model-based approaches in elucidating complex pharmacokinetic behaviors, improving exposure-response understanding, and facilitating the efficient early development of novel nanoparticle formulations.

## Data Availability

The original contributions presented in the study are included in the article/Supplementary Material, further inquiries can be directed to the corresponding author.
