# Supplementary material for: Model-based prediction of nanoparticle and dissolved form ratios using total concentration data: a case study of SNB-101
Source: Front Pharmacol. 2025 Aug 21;16:1556618. doi: 10.3389/fphar.2025.1556618 (PMC12408558; doi:10.3389/fphar.2025.1556618)
Supplement: Supplementary file 1 [file DataSheet1.docx]

**Model Code**

$PROB SNB101 NP_P

$ABBR DERIV2=NO

$INPUT ID TIME AMT RATE DV MDV CMT CYCLE BSA COHORT DOSE TAD DUR IND

$DATA ..//snb_dataset7_NP.csv IGNORE=@

$SUBROUTINE ADVAN13 TOL=5

$MODEL

COMP=(1.IRI_N_CENT)

COMP=(2.IRI_N_PERI)

COMP=(3.IRI_S_CENT)

COMP=(4.IRI_S_PERI1)

COMP=(5.IRI_S_PERI2)

COMP=(6.SN_N_CENT)

COMP=(7.SN_N_PERI)

COMP=(8.SN_S_CENT)

COMP=(9.SN_S_PERI)

COMP=(10.SN_G_CENT)

COMP=(11.SN_G_PERI)

$PK

TVCL1 = THETA(1) ;CL OF IRI_S

TVV1 = THETA(2) ;IRI_N

TVV2 = THETA(3) ;IRI_N

TVQ1 = THETA(4) ;IRI_N

TVK13 = THETA(5) ;IRI_N TO IRI_S

TVV3 = THETA(6) ;IRI_S

TVV4 = THETA(7) ;IRI_S

TVV5 = THETA(8) ;IRI_S

TVQ2 = THETA(9) ;IRI_S

TVQ3 = THETA(10) ;IRI_S

TVK68 = THETA(11) ;SN_N TO SN_S

TVV8 = THETA(12) ;SN_S

TVV9 = THETA(13) ;SN_S

TVQ5 = THETA(14) ;SN_S

TVCL2 = THETA(15) ;SN TO SNG

TVFMS = THETA(16) ;FRACTION SNS TO SNG

TVCL3 = THETA(17)

TVQ6 = THETA(18)

TVV10 = THETA(19) ; SNG

CL1 = TVCL1 * EXP(ETA(1))

V1 = TVV1 * EXP(ETA(2))

V2 = TVV2 * EXP(ETA(3))

Q1 = TVQ1 * EXP(ETA(4))

K13 = TVK13 * EXP(ETA(5))

V3 = TVV3 * EXP(ETA(6))

V4 = TVV4 * EXP(ETA(7))

V5 = TVV5 * EXP(ETA(8))

Q2 = TVQ2 * EXP(ETA(9))

Q3 = TVQ3 * EXP(ETA(10))

K68 = TVK68 * EXP(ETA(11))

V8 = TVV8 * EXP(ETA(12))

V9 = TVV9 * EXP(ETA(13))

Q5 = TVQ5 * EXP(ETA(14))

CL2 = TVCL2 * EXP(ETA(15))

FMS = TVFMS * EXP(ETA(16))

CL3 = TVCL3 * EXP(ETA(17))

Q6 = TVQ6 * EXP(ETA(18))

V10 = TVV10 * EXP(ETA(19))

V6 = V1 ;VC OF NANOPARTICLE

V7 = V2 ;VP OF NANOPARTICLE

Q4 = Q1 ; Q OF NANOPARTICLE

V11 = V9

S1 = V1/1000 ;AMT (umol) DV (nmol/L)

S3 = V3/1000

S6 = V6/1000

S8 = V8/1000

S10 = V10/1000

K12 = Q1/V1

K21 = Q1/V2

K34 = Q2/V3

K43 = Q2/V4

K35 = Q3/V3

K53 = Q3/V5

K30 = CL1*0.97/V3

K38 = CL1*0.03/V3

K67 = Q4/V6

K76 = Q4/V7

K89 = Q5/V8

K98 = Q5/V9

K80 = CL2*(1-FMS)/V8

K810 = CL2*FMS/V8

K100 = CL3/V10

K1011 = Q6/V10

K1110 = Q6/V11

$DES

DADT(1) = -(K13+K12)*A(1)+K21*A(2)

DADT(2) = -K21*A(2)+K12*A(1)

DADT(3) = -(K34+K35+K38+K30)*A(3)+K13*A(1)+K43*A(4)+K53*A(5)

DADT(4) = -K43*A(4)+K34*A(3)

DADT(5) = -K53*A(5)+K35*A(3)

DADT(6) = -(K67+K68)*A(6)+K76*A(7)

DADT(7) = -K76*A(7)+K67*A(6)

DADT(8) = -(K89+K80+K810)*A(8)+K38*A(3)+K68*A(6)+K98*A(9)

DADT(9) = -K98*A(9)+K89*A(8)

DADT(10) = K810*A(8)-(K100+K1011)*A(10)

DADT(11) = K1011*A(10) - K1110*A(11)

SNS = A(8)/S8

SNNP = A(6)/S6

$ERROR

IF(IND.EQ.1) THEN

IPRED = A(1)/S1 + A(3)/S3

;IPRED = A(3)/S3

W = SQRT(THETA(20)**2 + THETA(21)**2*IPRED**2)

IRES = DV - IPRED

IWRES = IRES / W

ENDIF

IF(IND.EQ.2) THEN

IPRED = A(6)/S6 + A(8)/S8

W = SQRT(THETA(22)**2 + THETA(23)**2*IPRED**2)

IRES = DV - IPRED

IWRES = IRES / W

ENDIF

IF(IND.EQ.3) THEN

IPRED = A(10)/S10

W = SQRT(THETA(24)**2 + THETA(25)**2*IPRED**2)

IRES = DV - IPRED

IWRES = IRES / W

ENDIF

Y = IPRED + W * EPS(1)

$THETA

(0, 30) ; 1. CL1 IRI_S

(0, 100) ; 2. V1 IRI_N

(0, 1000) ; 3. V2 IRI_N

(0, 15) ; 4. Q1 IRI_N

(0, 100) ; 5. K13 IRI N TO S

68.6 FIX ; 6. V3

67.2 FIX ; 7. V4

127 FIX ; 8. V5

114 FIX ; 9. Q2

9.89 FIX ; 10. Q3

(0, 1) ; 11. K68 SN N TO S

(0, 50) ; 12. V8 50

(0, 400) ; 13. V9

(0, 500) ; 14. Q5

(0, 100) ; 15. CL2 1040 FIX

1 FIX ; 16.FMS

(0, 10) ; 17. CL3

(0, 10) ; 18.Q6

(0, 50) ; 19. V10

10; 0.00001 FIX ;20. Add, IRI

0.2 ;21. Prop, IRI

0.00001 FIX ;0.00001 FIX ;22. Add, SN

0.2 ;23. Prop, SN

10; 0.00001 FIX ;24. Add, SNG

0.2 ;25. Prop, SNG

$OMEGA

0.02 ; 1. CL1 IRI_S

0.02 ; 2. V1 IRI_N

0.02 ; 3. V2 IRI_N

0 FIX ; 4. Q1 IRI_N

0 FIX ; 5. K13 IRI N TO S

0.023302744 FIX ; 6. V3 FIX

0.025809644 FIX ; 7. V4 FIX

0.08134926 FIX ; 8. V5 FIX

0.554644021 FIX ; 9. Q2 FIX

0.21915561 FIX ; 10. Q3 FIX

0.02 ; 11. K68 SN N TO S

0 FIX ; 12. V8

0 FIX ; 13. V9

0 FIX ; 14. Q5

0.04 ; 15. CLS 1040 FIX

0 FIX ; 16.FMS

0.02 ; 17. CL3

0 FIX ; Q6

0.02 ; V10

;REF ETA

; 0.10099942 FIX ;CL_CPT11

; 0.41292058 FIX ;CL_SN38

; 0.63917451 FIX ;CL_SN38G

; 0.023302744 FIX ;V1 -> V3

; 0.025809644 FIX ;V2 -> V4

; 0.08134926 FIX ;V3 -> V5

; 0.745124641 FIX ;V4

; 0.343305976 FIX ;V5

; 0.595316026 FIX ;V6

; 0.409139018 FIX ;V7

; 0.554644021 FIX ;Q2

; 0.21915561 FIX ;Q3

; 0.106564397 FIX ;Q5

; 0.623207432 FIX ;Q7

$SIGMA

1 FIX

$ESTIMATION NOABORT MAXEVAL=9999 METHOD=1 INTER PRINT=5 SIGDIGITS=2

$COV PRINT=E MATRIX=S

$TABLE ID TIME AMT RATE DV MDV CMT CYCLE BSA COHORT DOSE TAD DUR IND SNS SNNP IPRED CWRES IWRES ONEHEADER NOPRINT FILE = sdtab1013

$TABLE ID TIME CL1 V1 V2 Q1 K13 V3 V4 V5 Q2 Q3 K68 V8 V9 Q5 CL2 FMS CL3 Q6 ETA1 ETA2 ETA3 ETA4 ETA5 ETA6 ETA7 ETA8 ETA9 ETA10 ETA11 ETA12 ETA13 ETA14 ETA15 ETA16 ETA17 ETA18 NOPRINT NOAPPEND ONEHEADER FILE=patab1013
